# Spatiotemporal Distribution Characteristics and Driving Factors of Phytoplankton in the Mainstream of the Yellow River (Shandong Section)

**DOI:** 10.3390/microorganisms14061351

**Published:** 2026-06-16

**Authors:** Jingjing Wei, Xiaomeng Tian, Shiqi Xu, Shan Jiang, Jielin Wei, Haiyan Pei

**Affiliations:** 1 Department of Environmental Science and Engineering, Fudan University, Shanghai 200433, China; 2 Shandong Provincial Engineering Center on Environmental Science and Technology, Jinan 250061, China; 3 Institute of Eco-Chongming (IEC), Shanghai 202162, China

**Keywords:** Lower Yellow River, diatoms, phytoplankton community, spatiotemporal variation, environmental drivers, redundancy analysis

## Abstract

To investigate the spatiotemporal distribution of phytoplankton communities and their major environmental influences in the mainstream of the Yellow River (Shandong section), 29 sampling sites were surveyed in the summer and autumn of 2022 and the spring of 2023. Among the 206 phytoplankton species belonging to 8 phyla and 99 genera, Bacillariophyta, Chlorophyta, and Cyanobacteria were the main dominant groups, and *Aulacoseira granulata var. angustissima* (O.Müller) Simonsen, *Fragilaria capucina* Desmazières, and *Ulnaria acus* (Kützing) Aboal were the most important dominant species. The number of phytoplankton species and cell densities exhibited significant seasonal changes in the sequence of summer > autumn > spring. The phytoplankton community underwent a succession from Bacillariophyta–Chlorophyta dominance in spring and summer to Bacillariophyta–Cyanobacteria dominance in autumn. Water temperature, dissolved oxygen, and transparency were the most important factors affecting the growth of the dominant Bacillariophyta species. The spatiotemporal distribution of phytoplankton is jointly regulated by multiple environmental factors, providing a scientific basis for the evaluation and management of regional aquatic ecosystems.

## 1. Introduction

As the second-longest river in China, the Yellow River plays an important role both in regional economic development and as an ecological barrier for northern China [1]. Since 1949, China has made remarkable achievements in flood control and sediment management in the Yellow River [2,3]. However, water-ecological monitoring in the basin has been lagging for a long time, resulting in persistent issues such as decreased biodiversity and increased ecological instability [4,5,6]. Therefore, it is necessary to conduct systematic surveys of the river’s water ecology and evaluate its condition to implement the policy of ecological protection and high-quality development in the Yellow River Basin [7,8,9].

Phytoplankton play an important role as primary producers in aquatic ecosystems. They are characterized by their small body size, rapid reproduction and high sensitivity to environmental changes, which make them reliable bioindicators of ecosystem conditions [10]. At present, most investigations carried out in the Yellow River Basin focus on the water chemistry [11,12] or on the wide range of biodiversity [13,14]. Although these studies offer certain advantages, they still have shortcomings. Firstly, existing research is largely concentrated on river estuaries, headwaters, and small tributaries, while systematic investigations of the mainstream remain scarce—especially in the Shandong section. As the final downstream reach before the Yellow River flows into the Bohai Sea, this section features distinct hydrological characteristics, including reduced flow velocity and complex sediment dynamics [4,5,6]. It serves as a critical water-supply corridor, a major flood-control zone, and an important ecological transition zone connecting freshwater riverine systems and coastal marine environments [2,3]. Affected by continuous terrestrial input and sediment accumulation, its aquatic ecosystem is highly vulnerable to environmental fluctuations [2,3]. Secondly, the methods used are primarily based on traditional taxonomic surveys. We do not have sufficient data on the spatial and temporal variations in phytoplankton community structure and their responses to major environmental factors, such as nitrogen, phosphorus, and hydrological conditions [15,16,17].

To fill this gap, this study concentrates on the mainstream of the Yellow River (Shandong section). By means of year-round continuous observation, our objectives are: (1) to clarify the spatiotemporal variations in phytoplankton community structure, density, and the dominant species; and (2) to reveal how key environmental variables (including water temperature, nutrients and turbidity) regulate the growth and distribution of dominant phytoplankton species.

The results will offer scientific support for evaluating the aquatic ecological condition in the mainstream of the Yellow River (Shandong section) and for its ecological management. Meanwhile, this work will supplement the phytoplankton research database for this typical downstream river reach and enrich relevant ecological studies of the entire Yellow River Basin.

## 2. Materials and Methods

### 2.1. Sampling Period and Site Layout

**Figure 1:**
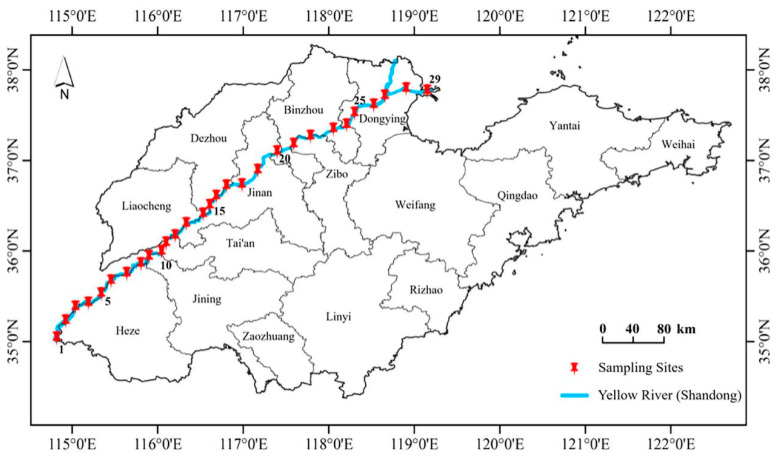
Sampling sites along the mainstream of the Yellow River in Shandong.

The mainstream of the Yellow River (Shandong section) runs westward from Dongming County, Heze City, Shandong Province, passing through Heze, Jining, Tai’an, Liaocheng, Dezhou, Jinan, Zibo, Binzhou, and Dongying, and terminates at the Yellow River estuary in Kenli District, Dongying City. It flows in a north–northeast direction, covering approximately 628 km and a watershed area of 18,300 km^2^, accounting for about 11.6% of the province’s total area [18]. According to the environmental features of the mainstream (Shandong section) and following the principles of representativeness, systematicness, and comprehensiveness specified in HJ 91.2-2022 [19] and the phytoplankton sampling guidelines from HJ 1215-2021 [20], this study established 29 sampling sites (Figure 1) sequentially along the river from its entry into Shandong Province to its estuary. The sampling sites were strategically selected to cover key hydrological sections (including the upper, middle, and lower reaches of the Shandong section), major tributary confluences, urbanized areas, water intake structures, and the estuarine transition zone to ensure comprehensive representation of different ecological zones and anthropogenic impact gradients in the study area. Sampling site 3 was not accessible during the summer sampling campaign due to a sudden water-level rise, which prevented our sampling vessel from reaching the designated location. Sampling was conducted in three seasons spanning two consecutive years: summer 2022, autumn 2022 and spring 2023.

### 2.2. Field Collection and Laboratory Analysis

The water depth at all sampling sites was measured during the field campaign and ranged from 1.0 m to 3.9 m across the three seasons, with an average depth of approximately 2.0 m. According to the Chinese national standard HJ 91.2-2022 [19], for water bodies with a depth of ≤5 m, sampling at a single depth of 0.5 m below the surface is sufficient and compliant. Water transparency was measured at each sampling site using a Secchi disk. 1 L of water sample was taken from the surface layer with a Ruttner standard water sampler (HYDRO-BIOS Apparatebau GmbH, Altenholz, Germany, with a capacity of 1.0 L). Light availability is a key factor regulating phytoplankton growth, and transparency has been extensively applied as an indicator of light availability in studies of phytoplankton ecology and community dynamics [21,22]. The water temperature (WT), dissolved oxygen (DO), and pH were measured immediately on-site with a portable, conventional five-parameter water-quality meter (BIO-5C, Shanghai Ronghui Instrument Technology Co., Ltd., China). The samples were then transferred to the laboratory, where the concentrations of total nitrogen (TN), ammonia nitrogen, and total phosphorus (TP) were determined. The procedures for measuring the laboratory water-quality parameters are given in Table 1 [23]. A portable current meter was used to measure the water velocity at each sampling point at a depth of 0.5 m below the water surface. Three duplicate readings were taken and averaged them. Velocity is used as the primary index of hydrological conditions because it directly affects residence time, mixing and potential resuspension of phytoplankton.

For quantitative analysis of phytoplankton, a 1 L water sample was collected to determine the qualitative and quantitative characteristics of phytoplankton. All samples were fixed on-site with Lugol’s solution [24]. After settling for 48 h, the phytoplankton samples were concentrated by removing the supernatant. The pellet was then resuspended and adjusted to a final volume of 10 mL [25]. The samples were observed and photographed under an optical microscope (CX31, Olympus, Japan) at 400× magnification. Phytoplankton species were identified, and cell densities were quantified using a hemocytometer. Taxonomic identification was primarily based on two authoritative Chinese monographs: *Flora Algarum Sinicarum Aquae Dulcis* and *Freshwater Algae of China: Systematics, Taxonomy and Ecology*, with additional reference to the relevant literature [26]. At least 200 algae individuals were identified for qualitative analysis in the continuous visual field of each sample. For the quantitative analysis, a known volume of well-mixed sample was pipetted onto a hemocytometer (1 mm × 1 mm × 0.1 mm), ensuring no air bubbles were present in the chamber. Two biological replicate water samples were collected at each sampling site. Each sample was counted three times and if the absolute deviation of the results of each two times was greater than 15% sampling and counting was continued. The average of three values that meet the above requirements was the result of quantitative analysis [27].

### 2.3. Data Analysis Methods

Pearson correlation analysis was performed using SPSS 26.0 (IBM Corp., Armonk, NY, USA) to examine the linear relationships between the dominant diatom species and environmental variables [28]. A significance level of *p* < 0.05 was used for all statistical tests.

Redundancy Analysis (RDA) was conducted using the “vegan” package (version 2.6-4) in Rstudio 4.1.3 to identify the key environmental factors driving the diatom community structure. Prior to RDA, all environmental data were log-transformed (log_10_(x + 1)) to reduce the influence of extreme values and improve normality. A detrended correspondence analysis (DCA) was first performed to determine the appropriate ordination method; the gradient length was less than 3 standard deviations, confirming that RDA was the most suitable method. The significance of the RDA model and each environmental factor was tested using Monte Carlo permutation tests with 999 permutations. This analytical framework has been widely used in previous phytoplankton studies in the Yellow River Basin [29].

## 3. Results

### 3.1. Analysis of Physicochemical Parameters

**Figure 2:**
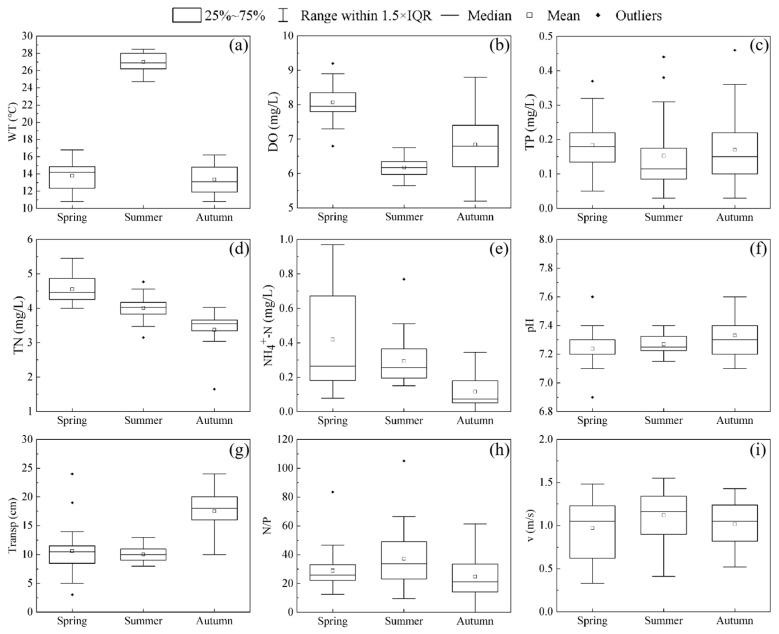
Seasonal variation in physicochemical factors at 29 sampling sites in the Yellow River. (**a**) WT = water temperature; (**b**) DO = dissolved oxygen; (**c**) TP = total phosphorus; (**d**) TN = total nitrogen; (**e**) NH_4_^+^–N = ammonium nitrogen; (**f**) pH; (**g**) Transp = transparency; (**h**) N/P = nitrogen-to-phosphorus ratio; (**i**) v = flow velocity. Spring, summer, and autumn correspond to spring 2023, summer 2022, and autumn 2022.

Figure 2 shows the seasonal variation in physicochemical parameters. Water temperature showed clear seasonal variations, with the summer temperatures being significantly higher than those in spring and autumn. The range of summer water temperature was 24.7–28.5 °C, whereas that in spring and autumn was 10.8–16.8 °C. Dissolved oxygen also varied seasonally, with the spring value (6.8–9.2 mg/L) being greater than that in autumn (5.2–8.8 mg/L), which in turn was greater than the summer value (5.7–6.8 mg/L). The pH values did not change greatly with the seasons, ranging from 6.9 to 7.6, indicating a slightly alkaline water body. Total nitrogen concentrations showed distinct seasonal changes, decreasing from spring (4.16–5.45 mg/L) to summer (3.15–4.77 mg/L) and to their lowest levels in autumn (0.69–4.02 mg/L). Ammonia nitrogen followed the same seasonal trend as the total nitrogen. Total phosphorus showed minimal seasonal variation, with concentrations ranging from 0.03 to 0.46 mg/L. The N/P ratio showed significant seasonal fluctuations, ranging from 12.41 to 83.40 in spring, reaching the widest range in summer (9.39–105.00) and then decreasing to 6.27–61.33 in autumn. The transparency showed distinct seasonal changes, with autumn values higher than those in spring and summer. The greatest variations occurred in spring, ranging from 3 to 24 cm; the least variations happened in summer, ranging from 8 to 13 cm; and in autumn, the range was 10 to 24 cm. The seasonal changes in flow velocity were not obvious, with the ranges being 0.33–1.48 m/s in spring, 0.52–1.55 m/s in summer, and 0.52–1.42 m/s in autumn. In general, water temperature, dissolved oxygen, and transparency each reached their seasonal highs in summer, spring, and autumn, respectively. Total nitrogen and ammonia nitrogen gradually decreased from spring to summer and autumn, whereas pH, total phosphorus, and flow velocity did not show significant seasonal variation.

One-way Analysis of Variance (ANOVA) followed by Tukey’s Honestly Significant Difference (HSD) test was performed to verify the statistical significance of these seasonal variations. The results showed that all measured physicochemical parameters except flow velocity (v) exhibited statistically significant seasonal differences (*p* < 0.05). Flow velocity showed no significant seasonal variation (*p* = 0.18). We speculate that this is mainly due to the stable hydrological regulation of the lower Yellow River: as an intensively managed alluvial river with numerous dams and sluices, the flow velocity of the Shandong section is artificially maintained at a relatively stable level throughout the year, resulting in no statistically significant seasonal difference.

### 3.2. Spatial Distribution Characteristics of Phytoplankton

A total of 206 phytoplankton taxa were identified across all 29 sampling sites, belonging to 8 phyla and 99 genera. Bacillariophyta (35.92%), Chlorophyta (33.50%) and Cyanobacteria (17.48%) were the three most species-rich groups, collectively accounting for 86.90% of the total species richness.

**Figure 3:**
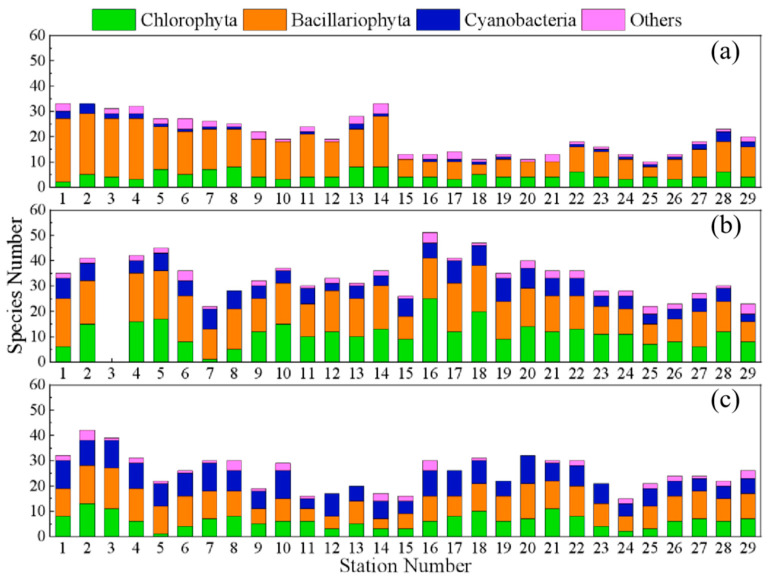
Distribution of phytoplankton species richness along the mainstream of the Yellow River in Shandong at 29 sampling sites across different seasons. (**a**–**c**) correspond to spring 2023, summer 2022, and autumn 2022, respectively.

As shown in Figure 3, the number of phytoplankton species showed significant seasonal changes, with the highest number in summer (22–51 species, average 34), followed by autumn (15–42 species, average 26) and then spring (10–33 species, average 21). The proportion of different phyla varied greatly in each season: Bacillariophyta and Chlorophyta were dominant in spring; in summer, the species of Chlorophyta and Cyanobacteria increased, but Bacillariophyta and Chlorophyta still dominated; and in autumn, the number of Cyanobacteria species remarkably increased, and Bacillariophyta and Cyanobacteria became the main groups.

**Figure 4:**
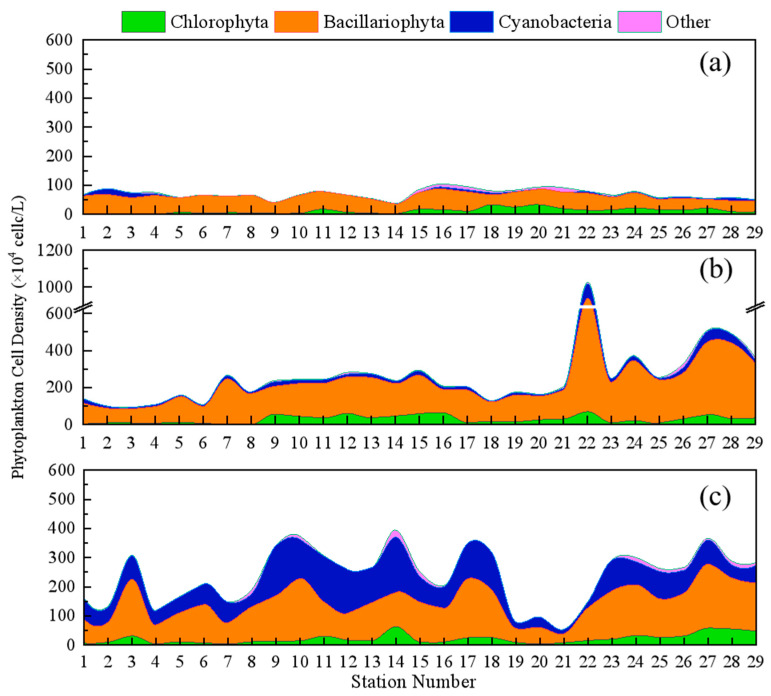
Variation in algal cell density in the mainstream of the Yellow River in Shandong. (**a**–**c**) correspond to spring 2023, summer 2022, and autumn 2022, respectively.

As depicted in Figure 4, the total phytoplankton cell density showed obvious seasonal changes, with values ranging from 100–1027 × 10^4^ cells/L in summer to 54–394 × 10^4^ cells/L in autumn and 38–104 × 10^4^ cells/L in spring. The density of different phyla also varied greatly among seasons. In spring, the average densities of Bacillariophyta, Chlorophyta, and Cyanobacteria were 52 × 10^4^ cells/L, 12 × 10^4^ cells/L, and 4 × 10^4^ cells/L, respectively, accounting for 74.4%, 16.8%, and 4.9% of the total. In summer, the average densities of Bacillariophyta, Chlorophyta, and Cyanobacteria were 216 × 10^4^ cells/L, 30 × 10^4^ cells/L, and 20 × 10^4^ cells/L, respectively, accounting for 79.7%, 11.4%, and 7.5% of the total. In autumn, the average densities of Bacillariophyta, Cyanobacteria, and Chlorophyta were 128 × 10^4^ cells/L, 85 × 10^4^ cells/L, and 22 × 10^4^ cells/L, respectively, accounting for 54.4%, 35.0%, and 8.7% of the total. The density of Bacillariophyta accounted for the largest proportion in all three seasons and was more than 50% in each. Except for autumn, the proportion of cyanobacteria in spring and summer was less than 10%.

### 3.3. Spatiotemporal Distribution Characteristics of Dominant Diatom Cell Densities

**Figure 5:**
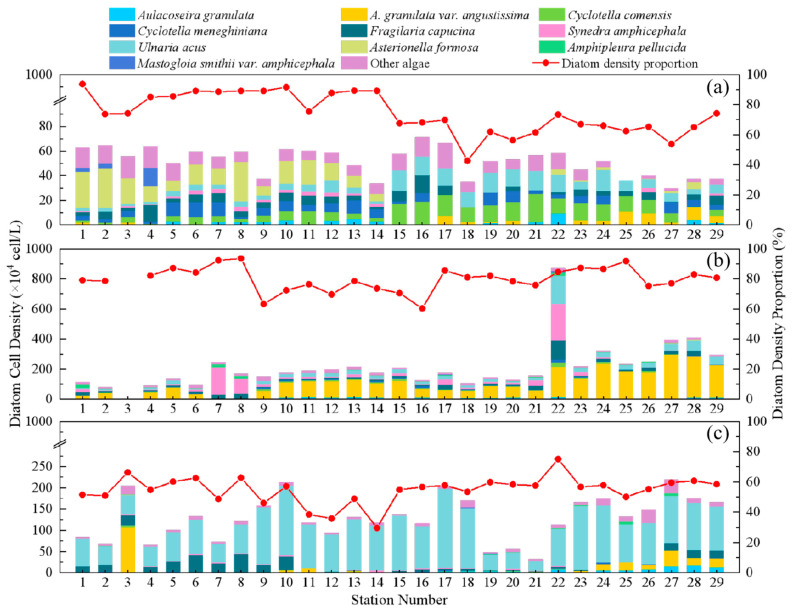
Variation in diatom density and the proportion of diatom density in the mainstream of the Yellow River in Shandong. (**a**–**c**) correspond to spring 2023, summer 2022, and autumn 2022, respectively.

As shown in Figure 5, the diatom cell density ranged from 30 to 868 × 10^4^ cells/L with an average of 130 × 10^4^ cells/L. The diatom cell density changed in the same seasonal pattern as the total phytoplankton cell density, changing from summer (79 to 868 × 10^4^ cells/L, mean 216 × 10^4^ cells/L) to autumn (31–218 × 10^4^ cells/L, mean 128 × 10^4^ cells/L) to spring (30–71 × 10^4^ cells/L, mean 52 × 10^4^ cells/L). On a spatial scale, the diatom cell density did not show much difference between adjacent sampling points in spring; however, in summer, the diatom cell density at sampling site 22 reached up to 868 × 10^4^ cells/L, while at sampling site 2 it was only 78 × 10^4^ cells/L, which indicates a significant variation along the river course and a trend of first increasing, then decreasing, and then increasing again. In autumn, diatom cell density was 278 × 10^4^ cells/L at sampling site 27 but only 32 × 10^4^ cells/L at sampling site 21, indicating a distinct variation along the river course. Bacillariophyta was the predominant phylum in each of the three seasons.

*Aulacoseira granulata var. angustissima* (O.Müller) Simonsen, *Fragilaria capucina* Desmazières and *Ulnaria acus* (Kützing) Aboal were predominant in all three seasons, whereas *Cyclotella comensis*. *Pantocsekiella comensis* (Grunow) K.T.Kiss & E.Ács, *Cyclotella meneghiniana*. *Stephanocyclus meneghinianus* (Kützing) Kulikovskiy, Genkal & Kociolek and Asterionella formosa Hassall were predominant only in spring, and *Aulacoseira granulata* (Ehrenberg) Simonsen, *Synedra amphicephala*. Fragilaria amphicephaloides Lange-Bertalot and *Amphipleura pellucida* (Kützing) Kützing were predominant only in summer.

### 3.4. Relationship Between Phytoplankton Community Structure and Environmental Factors

To investigate the responses of dominant diatoms to environmental factors, we selected the 10 most abundant diatom species for further analysis. These species collectively accounted for 82.9%, 94.7% and 94.6% of the total diatom cell density in spring 2023, summer 2022 and autumn 2022, respectively. Each species was dominant (relative abundance > 5%) in at least one season, ensuring they can effectively represent the core components of the diatom community and accurately reflect its seasonal succession characteristics. The selected species are *Aulacoseira granulata* (Ehrenberg) Simonsen, *Aulacoseira granulata var. angustissima* (O.Müller) Simonsen, and *Cyclotella comensis*. *Pantocsekiella comensis* (Grunow) K.T.Kiss & E.Ács, *Cyclotella meneghiniana*. *Stephanocyclus meneghinianus* (Kützing) Kulikovskiy, Genkal & Kociolek, *Fragilaria capucina* Desmazières, *Synedra amphicephala*. *Fragilaria amphicephaloides* Lange-Bertalot, *Ulnaria acus* (Kützing) Aboal, *Asterionella formosa* Hassall, *Amphipleura pellucida* (Kützing) Kützing, and *Mastogloia smithii var. Amphicephala* Grunow. Light micrographs of these ten dominant species are provided in Figure S1 (Supplementary Materials) for morphological reference.

According to Table 2, the main diatoms with a highly significant positive correlation (*p* < 0.001) with a correlation coefficient greater than 0.5 were: *Aulacoseira granulata var. angustissima* (O.Müller) Simonsen and water temperature; *Asterionella formosa* Hassall and dissolved oxygen; *Asterionella formosa* Hassall and ammonia nitrogen; and *Ulnaria acus* (Kützing) Aboal and transparency. The major diatom–environmental factor pairs exhibiting a highly significant negative correlation (*p* < 0.001) with a correlation coefficient over 0.5 were: *Aulacoseira granulata* (Ehrenberg) Simonsen and dissolved oxygen; *Ulnaria acus* (Kützing) Aboal and total nitrogen; and *Asterionella formosa* Hassall and flow velocity. It should be noted that multiple correlation tests were performed in this study, which may increase the risk of false positives. However, we mainly focused on strong correlations (correlation coefficients > 0.5 and *p* < 0.001), which have high reliability and low false-positive rates. These results are consistent with the subsequent redundancy analysis (RDA), further confirming their ecological significance.

Redundancy analysis (RDA) was performed to further explore the relationships between dominant diatom species and environmental factors. The Monte Carlo permutation test (999 permutations) confirmed that the RDA model was highly statistically significant (F = 6.5, *p* = 0.002 < 0.01). The first axis explained 19.14% of the total variance in diatom community structure and the second axis explained 17.10%, with a cumulative explanation rate of 36.24% for the first two axes.

**Figure 6:**
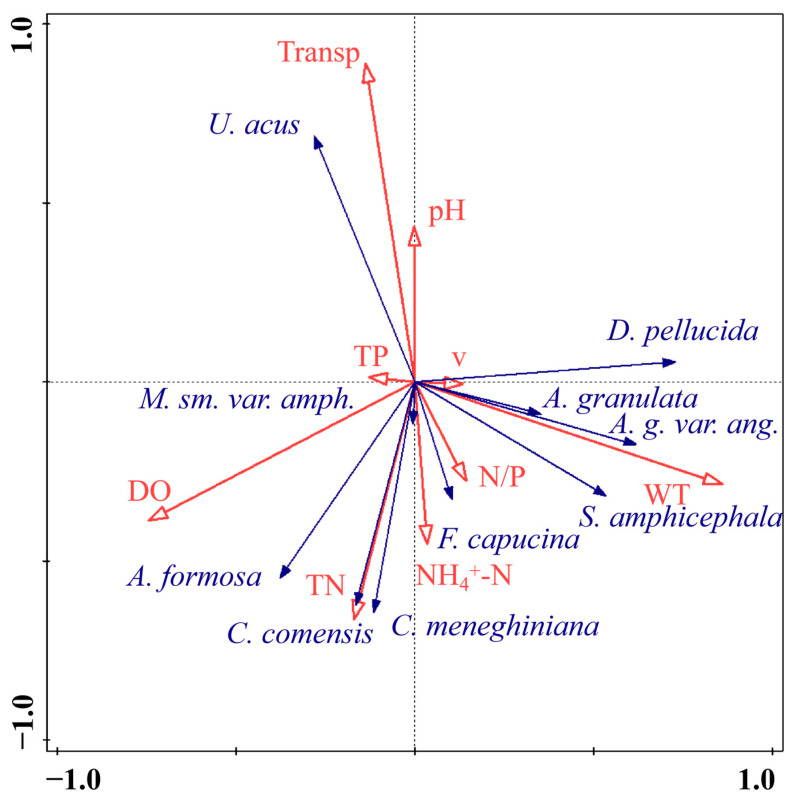
Redundancy analysis of dominant diatom communities and environmental factors.

As shown in Figure 6, water temperature, dissolved oxygen, and transparency are the three important environmental factors affecting the growth of the main diatom species. *Aulacoseira granulata var. angustissima* (O.Müller) Simonsen has significant positive correlations with water temperature and significant negative correlations with dissolved oxygen. *Ulnaria acus* (Kützing) Aboal exhibits significant positive correlations with transparency and pH, and significant negative correlations with total nitrogen and ammonia nitrogen. Conversely, *Fragilaria capucina* Desmazières has a significant negative correlation with transparency and pH, and a significant positive correlation with total nitrogen and ammonia nitrogen.

## 4. Discussion

### 4.1. Spatiotemporal Distribution Characteristics of the Phytoplankton Community

The phytoplankton community in the Yellow River (Shandong section) was dominated by Bacillariophyta, followed by Chlorophyta and Cyanobacteria. *Aulacoseira granulata var. angustissima* (O.Müller) Simonsen, *Fragilaria capucina* Desmazières, and *Ulnaria acus* (Kützing) Aboal were predominant in all three seasons. Wang et al. [30] identified 154 phytoplankton species in the Henan section of the Yellow River mainstream, belonging to 7 phyla and 65 genera. According to the species abundance, the order is Chlorophyta > Bacillariophyta > Cyanobacteria, with *Cyclotella* sp., *Aulacoseira granulata var. angustissima* (O.Müller) Simonsen, *Aphanizomenon* sp., and *Pseudanabaena* sp. being the dominant species. The phytoplankton in the mainstream of the Yellow River (Shandong section) are mainly composed of Bacillariophyta, and the richness (species number in Henan section: 154; species number in Shandong section: 206) and density (average algae density in Henan section: 68.25 × 10^4^ cells/L; average algae density in Shandong section: 216 × 10^4^ cells/L) are much higher than those in the Henan section [30]. In addition, nine dominant species are found in the Yellow River in summer. There are significant differences in phytoplankton composition and dominant types between the two sections of the mainstream, which are closely tied to differences in nutrient availability, hydrological conditions, and water chemistry. In summer, the total nitrogen concentration in the Henan and Shandong sections of the Yellow River is high (Henan: 1.67–5.41 mg/L; Shandong: 3.15–4.77 mg/L), and the total phosphorus concentration is relatively sufficient (Henan: 0.05–0.089 mg/L; Shandong: 0.03–0.44 mg/L), which could provide sufficient nutrition for the growth of phytoplankton [30]. Based on hydrological conditions, the Henan section is a wandering river, with high water and sediment content; the river channel in the Shandong section is stable in shape, and the sediment concentration in the water body is lower [21]. In addition, the velocity of the Henan section is relatively high, with an average velocity exceeding 2.5 m/s [31,32]. The average flow velocity across all sites and seasons was 1.12 m/s. Less sediment concentration and lower flow velocity are beneficial to the growth of algae [33,34], so the number of phytoplankton species, dominant species and total algae density in the Shandong section are higher than those in the Henan section. These environmental differences, including hydrological conditions, nutrient levels, and physical disturbances, have influenced algal sedimentation rates, light-use efficiency, and nutrient competition, resulting in significant difference in algal species composition between Henan and Shandong.

In the mainstream of the Yellow River (Shandong section), the species richness and density of phytoplankton are greater in summer than in autumn and spring. This is due to the high water temperature, high light intensity, and abundance of nitrogen and phosphorus in summer, which promote algal metabolism and photosynthesis, resulting in rapid algal growth and reproduction and therefore increasing algal abundance and density [35,36,37]. From spring to autumn, the phytoplankton community shifted from a Bacillariophyta–Chlorophyta assemblage to a Bacillariophyta–Cyanobacteria assemblage. The community structure shifted towards Cyanobacteria dominance as water temperature increased [38,39]. Cyanobacteria are generally thermophilic, growing extensively at temperatures above 20 °C [40]. In this research, summer water temperatures exceeded 20 °C, a range that promotes cyanobacterial growth and reproduction.

In spring, total phytoplankton density and species number showed a slight decline along the river, which may be due to the rapid downstream flow, leading to the dispersal or loss of nutrients and decreased transparency, thereby hindering algal growth [41]. Lakshmikandan et al. [42] stated that the number of phytoplankton species and biomass were largest in small water bodies. Liu et al. [43] also suggested that water conditions with low flow velocity, temperature, and light intensity, which are favorable for diatom growth under low temperature, low turbidity, and slight movement, resulted in diatoms as the dominant species. In this study, the low water flow rate at sampling sites 1–10 led to a high number of phytoplankton species and a high density in spring.

### 4.2. Major Environmental Factors Affecting the Growth of Dominant Diatom Species

Among the main phytoplankton species in the mainstream of the Yellow River (Shandong section) during summer, *Aulacoseira granulata* (Ehrenberg) Simonsen, *Aulacoseira granulata var. angustissima* (O.Müller) Simonsen, and *Amphipleura pellucida* (Kützing) Kützing have significant associations with water temperature and dissolved oxygen: all of them show a positive correlation with water temperature and a negative correlation with dissolved oxygen. *Aulacoseria* is a mesophyte diatom. High temperature enhances the activity of metabolic enzymes and the cell division rate in *Aulacoseria* species. In summer, the water residence time increases in the lower Yellow River, leading to sufficient thermal accumulation that provides favorable thermodynamic conditions for the proliferation of these diatoms [44,45]. The observed decline in dissolved oxygen results from two complementary mechanisms. First, high temperatures reduce the physical solubility of oxygen in water; previous studies have shown that water temperature is the dominant factor controlling dissolved oxygen levels in the Yellow River estuary during summer, with apparent oxygen consumption reaching 2.04 mL/L [46]. Second, dissolved oxygen depletion is also closely associated with nocturnal algal respiration and microbial decomposition of dead algal biomass [47]. Therefore, the significant positive correlation between Aulacoseira abundance and water temperature, and the significant negative correlation with dissolved oxygen, reflect a sequential ecological process: elevated water temperature stimulates diatom growth, which in turn increases biological oxygen consumption. Due to limitations in field conditions, hydrological conditions (such as water-level fluctuations and turbulence intensity), biological interactions (such as zooplankton grazing), and silica limitation were not detected in this study. Future research should include comprehensive monitoring to fully clarify the influence of hydrology and biological interactions on phytoplankton dynamics.

Therefore, the strong relationship between the dominant phytoplankton species and environmental conditions in the mainstream of the Yellow River (Shandong section) is due to the combined effects of several environmental factors, including water temperature, dissolved oxygen, nitrogen nutrients, transparency, and pH. This phenomenon can be explained by niche differentiation theory, which is driven by environmental heterogeneity [47,48,49]. Moreover, the large amounts of sediment and nutrient pulses brought by the Yellow River water and sediment regulation project have greatly changed the light environment and nutrient composition of the water body, thereby intensifying the dynamic succession of diatom communities [47]. These results provide insight into the formation mechanism of phytoplankton communities in the lower reaches of large rivers and serve as a biological basis for the comprehensive evaluation of water quality and the early detection of eutrophication risks in the mainstream of the Yellow River (Shandong section).

In addition to the conventional physicochemical indicators measured in this work, nitrogen and phosphorus, which we quantified, are the most dominant nutrient pollutants in the Yellow River (Shandong section). Excessive inputs of these nutrients are the primary drivers of eutrophication and phytoplankton blooms in riverine ecosystems. Furthermore, previous regional investigations have confirmed widespread coexistence of other typical pollutants, including organochlorine pesticides and polycyclic aromatic hydrocarbons, in this reach [50]. These exogenous pollutants can alter enzyme activity and interspecific competition among phytoplankton, further reshaping community succession. Due to the limitation of the field sampling design in this campaign, synchronous quantitative detection of heavy metals and organic pollutants was not carried out in our present survey.

### 4.3. Study Limitations

Two important limitations of this study should be acknowledged. First, this study primarily focused on the effects of key abiotic environmental factors on phytoplankton community structure, and did not systematically investigate other important ecological drivers: additional abiotic factors, including silica limitation and hydrological disturbances, as well as biotic factors, including grazing pressure from protozoa and metazoan zooplankton, shading effects, and allelopathic influences of macrophytes. Second, routine pollutant indices such as heavy metals and organic pollutants were not sampled and quantified alongside phytoplankton and basic water parameters. However, previous studies in this region have consistently demonstrated that the abiotic factors we focused on are the dominant drivers of phytoplankton dynamics, accounting for more than 50% of the total variation in community structure [51]. Future studies should measure dissolved silicon concentrations, conduct long-term hydrological monitoring, simultaneously investigate zooplankton community composition and quantify grazing pressure, combine macrophyte distribution surveys, and integrate synchronous pollutant monitoring to clarify the combined effects of conventional environmental factors and pollutant stress on phytoplankton dynamics, so as to comprehensively reveal the multi-factor coupled regulatory mechanisms of phytoplankton succession in this region.

## 5. Conclusions

This research carried out a comprehensive investigation of 29 sampling sites along the mainstream of the Yellow River (Shandong section) on an annual basis, examined the community structure of phytoplankton and its association with environmental factors, and summarized the following main findings:

(1) The phytoplankton community in the mainstream of the Yellow River (Shandong section) comprises 8 phyla, 99 genera and 206 species, with Bacillariophyta as the absolute dominant group throughout the year, followed by Chlorophyta and Cyanobacteria. *Aulacoseira granulata var. angustissima* (O.Müller) Simonsen, *Fragilaria capucina* Desmazières, and *Ulnaria acus* (Kützing) Aboal were the most important dominant species. All three seasons showed that Bacillariophyta was the absolutely dominant phylum, and it accounted for more than 50% of the density in each season.

(2) The number of phytoplankton species and cell density also showed significant seasonal variations: summer > autumn > spring. Regarding seasonal succession, the community shifted from Bacillariophyta–Chlorophyta dominance in spring and summer to Bacillariophyta–Cyanobacteria dominance in autumn, and Cyanobacteria gained dominance in autumn due to their buoyancy regulatory mechanisms.

(3) Water temperature, dissolved oxygen, and transparency are the main environmental conditions affecting the growth of the dominant diatoms. *Aulacoseira granulata* (Ehrenberg) Simonsen, *Aulacoseira granulata var. angustissima* (O.Müller) Simonsen, and *Amphipleura pellucida* (Kützing) Kützing showed significant positive relationships with water temperature and negative relationships with dissolved oxygen, reflecting the mechanism whereby high temperature stimulates algal growth and respiration, thereby consuming oxygen.

(4) The spatial and temporal variation in the phytoplankton community in the mainstream of the Yellow River (Shandong section) is due to the combined influences of several environmental factors such as water temperature, dissolved oxygen, nitrogen and phosphorus nutrients, transparency, and hydrodynamics. The results provide a scientific basis and biological support for evaluating aquatic ecological conditions, forecasting eutrophication risks, and implementing ecological regulation and management in this area.

## Figures and Tables

**Table 1:** Water-quality parameter measurement methods.

Water-Quality Parameter	Determination Method
TN	Alkaline potassium persulfate digestion–ultraviolet spectrophotometry
TP	Potassium persulfate digestion–molybdenum–antimony anti-color spectrophotometry
NH_4_^+^-N	Bromophenol blue method

**Table 2:** Pearson correlation analysis results between ten dominant diatom species and environmental factors.

	*A. gran.*	*A. g. var. ang.*	*C. come.*	*C. mene.*	*F. capu.*	*S. amph.*	*U. acus*	*A. form.*	*Amphiple.*	*M. sm. var. amph.*
WT	0.405 ***	0.646 ***	0.173	0.139	0.255 *	0.380 ***	−0.248 *	−0.351 ***	0.411 ***	0.002
DO	−0.525 ***	−0.416 ***	0.148	0.125	−0.223 *	−0.276 *	−0.387 ***	0.551 ***	−0.322 **	0.029
TP	−0.023	−0.124	0.041	0.004	−0.145	−0.033	0.085	0.123	−0.146	0.003
TN	−0.070	−0.031	0.302 **	0.314 **	−0.070	−0.018	−0.529 ***	0.428 ***	−0.138	0.123
NH_4_^+^-N	0.009	−0.012	0.120	0.324 **	−0.133	−0.008	−0.418 ***	0.687 ***	−0.006	0.183
pH	0.018	−0.020	−0.222 *	−0.133	−0.038	0.007	0.347 **	0.006	0.025	−0.234 *
Transp	−0.049	−0.255 *	−0.408 ***	−0.290 **	−0.077	−0.133	0.564 ***	−0.078	−0.034	−0.102
N/P	−0.027	0.184	0.067	0.091	0.134	0.045	−0.334 **	0.003	0.134	0.005
v	0.347 **	0.219 *	0.306 **	0.133	0.080	0.162	0.224 *	−0.535 ***	0.006	−0.110

Note: WT = Water temperature (°C); DO = dissolved oxygen (mg/L); TP = total phosphorus (mg/L); TN = total nitrogen (mg/L); NH_4_^+^-N = ammonium (mg/L); Transp = transparency (cm); N/P = nitrogen-to-phosphorus ratio; v = flow velocity (m/s); *A. gran.* = *Aulacoseira granulata* (Ehrenberg) Simonsen abundance (cells/L); *A. g. var. ang.* = *Aulacoseira granulata var. angustissima* (O.Müller) Simonsen abundance (cells/L); *C. come.* = *Cyclotella comensis. Pantocsekiella comensis* (Grunow) K.T.Kiss & E.Ács abundance (cells/L); *C. mene.* = *Cyclotella meneghiniana. Stephanocyclus meneghinianus* (Kützing) Kulikovskiy, Genkal & Kociolek abundance (cells/L); *F. capu.* = *Fragilaria capucina* Desmazières abundance (cells/L); *S. amph.* = *Synedra amphicephala. Fragilaria amphicephaloides* Lange-Bertalot abundance (cells/L); *U. acus* = *Ulnaria acus* (Kützing) Aboal abundance (cells/L); *A. form.* = *Asterionella formosa* Hassall abundance (cells/L); *Amphiple.* = *Amphipleura pellucida* (Kützing) Kützing abundance (cells/L); *M. sm. var. amph.* = *Mastogloia smithii var. Amphicephala* Grunow abundance (cells/L). *** denotes *p* < 0.001 (two-tailed); ** denotes *p* < 0.01 (two-tailed); * denotes *p* < 0.05 (two-tailed).

## Data Availability

Data will be made available on request.

## Supplementary Materials

The following supporting information can be downloaded at https://www.mdpi.com/article/10.3390/microorganisms14061351/s1. Figure S1. Light micrographs of the ten dominant diatom species in the mainstream of the Yellow River (Shandong section).
