# Supplementary material for: Spatiotemporal Distribution Characteristics and Driving Factors of Phytoplankton in the Mainstream of the Yellow River (Shandong Section)
Source: Microorganisms. 2026 Jun 16;14(6):1351. doi: 10.3390/microorganisms14061351 (PMC13304030; doi:10.3390/microorganisms14061351)
Supplement: Supplementary file 1 [file microorganisms-14-01351-s001.zip › microorganisms-4348047-supplementary.pdf]

Figure S1. Light micrographs of the ten dominant diatom species in the mainstream of the Yellow River (Shandong section)

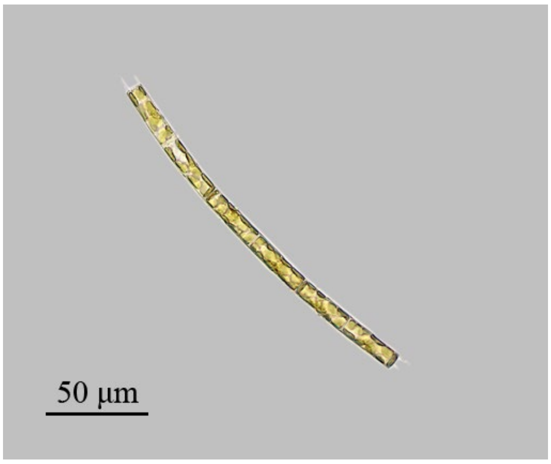

Aulacoseira granulata (Ehrenberg) Simonsen

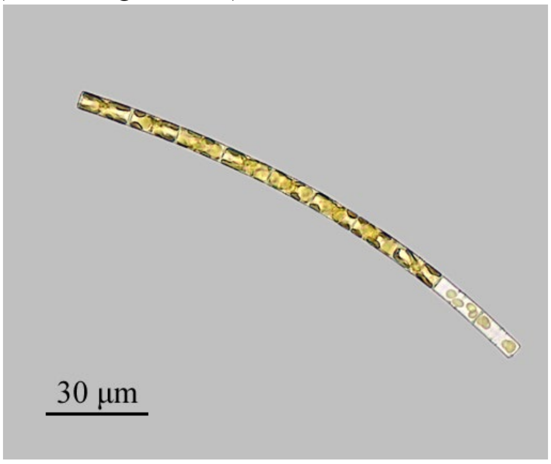

Aulacoseira granulata var. angustissima (O.Müller) Simonsen

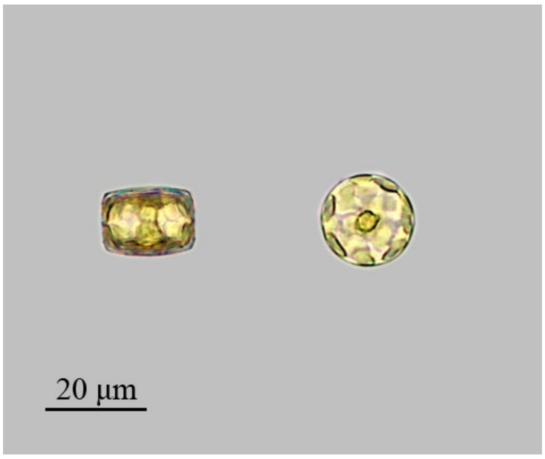

Cyclotella comensis. Pantocsekiella comensis (Grunow) K.T.Kiss & E.Ács

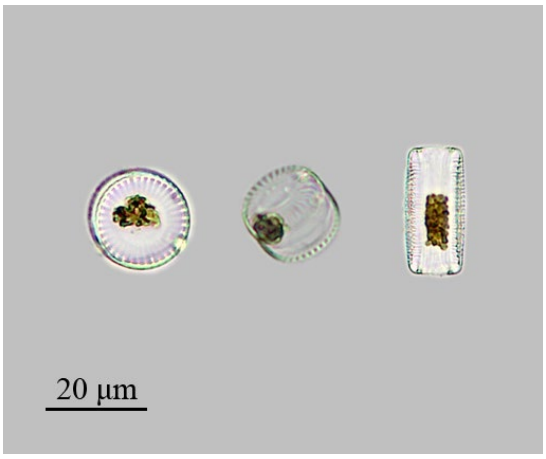

Cyclotella meneghiniana. Stephanocyclus meneghinianus (Kützing) Kulikovskiy, Genkal & Kociolek

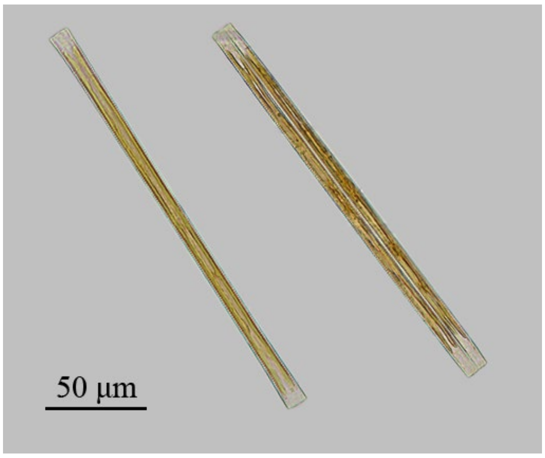

Fragilaria capucina Desmazières

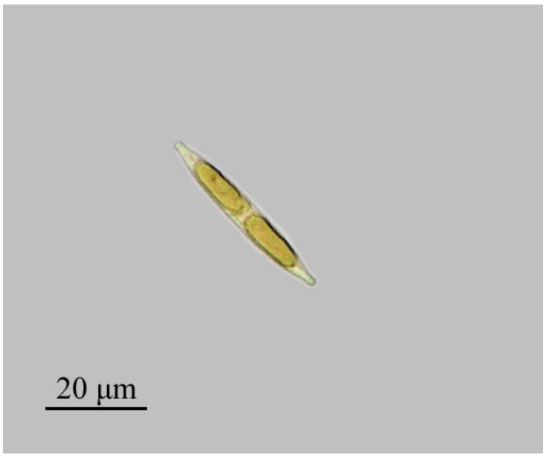

Synedra amphicephala. Fragilaria amphicephaloides Lange-Bertalot

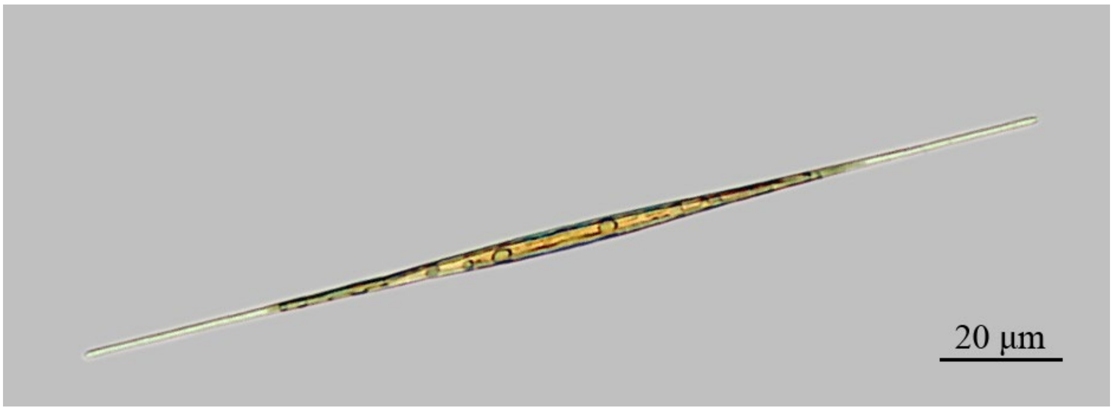

Ulnaria acus (Kützing) Aboal

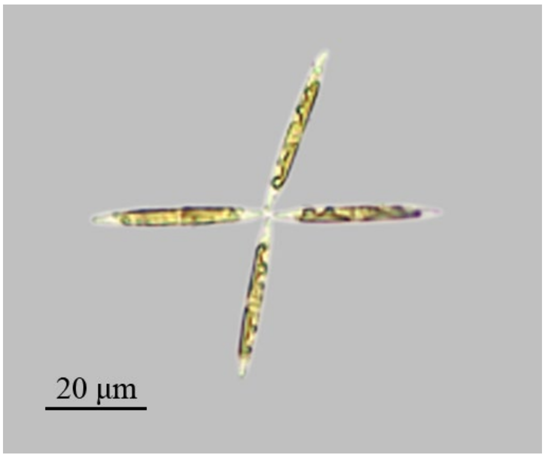

Asterionella formosa Hassall

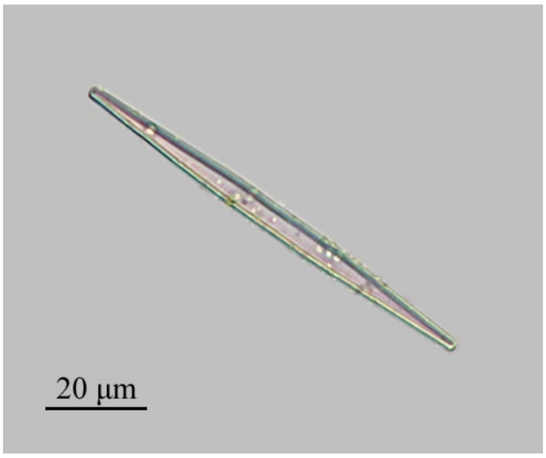

Amphipleura pellucida (Kützing) Kützing

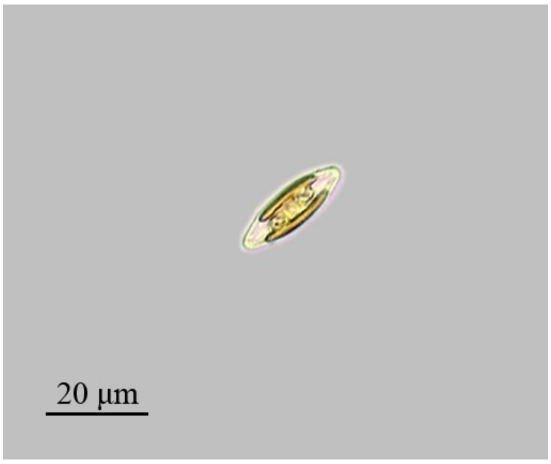

Mastogloia smithii var. Amphicephala Grunow
